# Assessing the Value of Testate Amoebae and their Functional Traits in Detecting Climate Change-Induced Peatland Drying

**DOI:** 10.1007/s00248-025-02682-2

**Published:** 2025-12-20

**Authors:** Olivia Kuuri-Riutta, Brunella Palacios Ganoza, Henni Ylänne, Edward A. D. Mitchell, Minna M. Väliranta, Eeva-Stiina Tuittila

**Affiliations:** 1 School of Forest Sciences, University of Eastern Finland, Joensuu, Finland; 2 Laboratory of Soil Biodiversity, University of Neuchâtel, Neuchâtel, Switzerland; 3 Ecosystemes and Environment Research Programme, Environmental Change Research Unit, University of Helsinki, Helsinki, Finland

**Keywords:** Testate amoeba ecology, Microbial community, Peatland microbial ecology, Paleo proxy, Resistance

## Abstract

Climate change-induced drying is projected for northern peatlands, but evidence on its extent is inconsistent. Testate amoebae (TA) are a water table depth proxy that also responds to vegetation succession and abiotic changes. However, it is not well-known how TA communities and functional traits differ between unaffected and drying areas in different peatland types. Thus, we compared TA communities and functional traits in undrained control areas and areas (initially similar in vegetation and WT) subjected to moderate water level drawdown (WLD) for ∼20 years. The experiment covers a rich fen, a poor fen, and a bog. Arboreal vegetation has been established in the fen WLD areas. Taxonomic composition and functional traits differed between the WLD and control, most notably in the rich fen and the least in the bog, mirroring the vegetation. Eleven taxa favored WLD or pristine conditions; six taxa had a site-specific preference. Small tests and apertures, heterotrophy, and siliceous tests were more common in the WLD than control areas, but in the bog, these differences were not significant. Overall, besides drying, the establishment of arboreal vegetation and changes in abiotic conditions affected TA community and trait compositions in the fens, showing that they deliver information not only about water table depth but also sensitivity/resistance of peatlands. When aiming for quantitative water table reconstructions, account should be taken that the secondary changes amplify the signal of WLD in fens, whereas the resistance of vegetation and testate amoeba communities may hinder it in bogs, possibly causing bias when the reconstruction runs through a fen-bog-transition.

**Supplementary Information:**

The online version contains supplementary material available at 10.1007/s00248-025-02682-2.

## Introduction

Peatlands are ecosystems characterized by high water table (WT) that slows down decomposition, allowing peatlands to sequester and store globally significant amounts of carbon [1]. Due to drastic warming and associated increase in evapotranspiration in high latitudes [2] temperate and boreal peatlands are becoming drier [3, 4]. As high WT is a key factor preventing the growth of shrubs and trees in peatlands, drying leads to the loss of peatland-specific vegetation and establishment of a tree stand, and further changes in abiotic conditions near the peatland surface [5,6]. This vegetation transition has been shown to vary in magnitude among peatland types, being the greatest in nutrient-rich sites [e.g., 5]. However, the extent of this ongoing drying and associated vegetation turnover is still poorly understood. Because long-term records of water table depth are scarce and typically cover a relatively short period, indirect proxies that provide a reference beyond the observation history are needed. Moreover, detecting ongoing drying needs to account the direct and indirect changes in the entire ecosystem.

A commonly used proxy for peatland WT is testate amoebae (TA), unicellular shelled protists whose taxa have well-defined optima (preferences) for water table depth in peatlands [7]. Their tests (shells) are preserved in peat from which they can be extracted and identified under a microscope. Early on, TA were classified according to their moisture preferences [7, and references therein], and extensive training sets that connect TA community composition to water table depth have shed more light on the hydrological preferences of TA [e.g., 8, 9]. Their sensitivity to variation in WT depth has been shown in short-term experiments covering a few years [e.g., 10, 11], and comparative field studies have shown that prevailing vegetation, peatland type, nutrient availability, peatland degradation, and exposure to soil frost also affect TA communities [e.g., 12, 13, 14]. However, it is not fully understood what kind of TA communities will occupy peatlands subjected to climate-induced drying, i.e., drying with moderate intensity that persists for decades, and how these differ along the nutrient gradient.

The traditional, taxonomy-based TA proxy includes certain limitations, such as inconsistent identification and nomenclature of TA taxa [15]. This has promoted the application of functional traits (hereafter abbreviated as “traits”) to the context of TA [16]. Traits are properties that reflect survival, development, and growth strategies in an individual organism and, thus, the environmental pressures the community is facing [17]. Changes in hydrology affect the trait composition of TA communities: dry and disturbed conditions generally favor small and compressed tests, and small and hidden apertures [e.g., 11, 16]. Proteinaceous tests and mixotrophs thrive in wet and open peatlands, while siliceous tests are more common in drier and forested conditions [18–20]. However, similarly to the species composition, it is not well-known how climate-induced drying affects TA traits.

Our aim is to assess the use of TA as a proxy for climate-induced drying and associated changes in boreal peatlands. We compare testate amoeba communities and functional traits between undrained control areas and water level drawdown (WLD) areas, where WT has been moderately lowered for two decades to simulate the impacts of climate-induced drying. The counterparts originally resembled each other in their vegetation and WT. The experiment covers three peatland types (rich fen, poor fen, and bog). We hypothesize that TA community composition and traits differ between control and WLD areas depending on site fertility, so that the largest contrasts between the control and the WLD area are displayed in the nutrient-rich fen and the smallest in the bog. To enhance the understanding on the ecological controls driving TA communities, we address the following research questions:

Q1: What are the water table optima of the TA taxa with significant preference for control or WLD conditions?

Q2: What are the environmental variables that explain the patterns observed in the taxa and traits?

## Materials and Methods

### Study Site

**Fig. 1:**
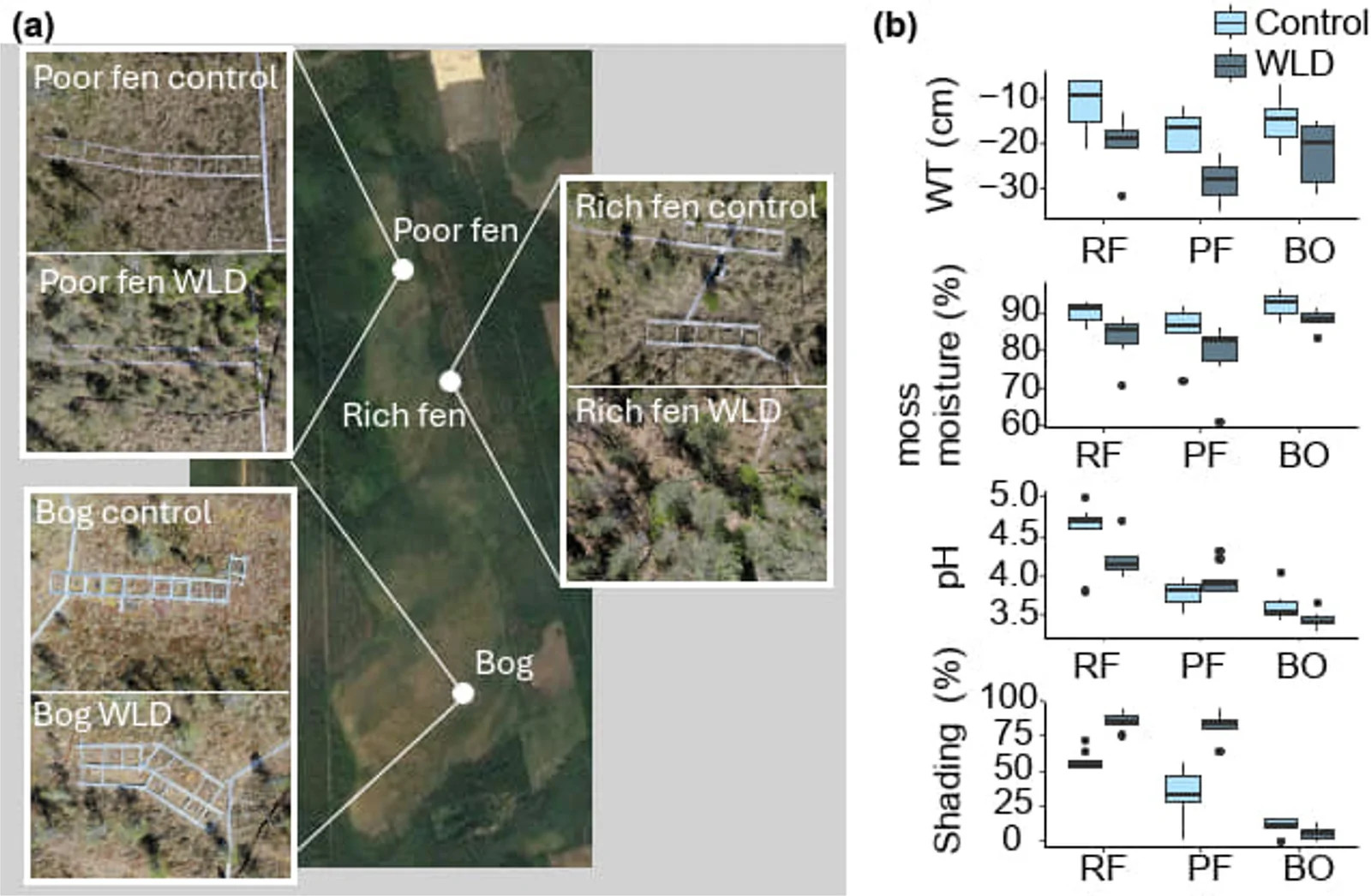
(**a**) Aerial photographs (taken by Pasi Jalkanen in 2023 and previously published in Köster *et al*., 2023 [22] of the six study areas and their locations within Lakkasuo peatland. (**b**) Environmental variables in Lakkasuo study areas [5, 21]. Negative WT values indicate water table below peat surface. RF = rich fen, PF = poor fen, BO = bog

Our study site, Lakkasuo (Fig. 1a), is an eccentric raised peatland complex in Orivesi, Central Finland (61°47′N, 24°18′E). Lakkasuo peatland complex consists of several peatland types, including a mesotrophic fen, an oligotrophic fen, and an ombrotrophic bog (hereafter referred to as rich fen, poor fen and bog, respectively). In three study sites representing these peatland types, a long-term water level drawdown (WLD) experiment was established in 2000–2001 to simulate climate-induced drying and the experiment is still ongoing. The WLD was executed by digging 30 cm-deep ditches around three experimental WLD areas and establishing corresponding, undrained control areas upstream of each WLD area [5]. At the beginning of the experiment, vegetation and water table depth were similar within each site: in the rich fen control area and the rich fen WLD area, as well as in the two poor fen study areas and in the two bog study areas [5]. To date, WLD has led to the establishment of tree stands that increase shading and nutrient concentrations in the fen sites but not in the bog [5, 21]. In 2022, water tables were on average 9 cm lower in the WLD areas than in the control areas (Fig. 1b).

### Data Collection

To assess the impact of WLD on TA communities and traits, we collected the topmost 3 centimeters of 3–10 moss shoots from the immediate proximity of 8–10 permanent sampling plots in each study area (total *n* = 53) in summer 2022. We sampled the moss species dominant in the permanent sampling plots, that was *Sphagnum* spp. in all but two sampling plots in the rich fen WLD area (see Supplementary Table 1). The samples were stored in 15 ml of 4% formaldehyde.

We prepared the TA samples by shaking the sample for a minute in hand, and a minute in a Vortex shaker, after which the sample was poured through a 150 μm sieve. The samples were then centrifuged to remove excess liquid. We analyzed the samples under a light microscope at 200x and 400x. TA were identified to morphotype level, using as references Siemensma (2023) [23] and photos in McKeown *et al*., (2019) [24], and counted aiming at the total count of 150 individuals [25]. However, when identification to the morphotype level was not reached, we applied the grouping presented in Amesbury* et al*., (2016) [9]. *Phryganella paradoxa* was identified as uncertain (cf.), as Siemensma (2023) [23] warns that the identification cannot be confirmed without inspecting pseudopodia.

The functional traits inspected were biovolume (based on test length and width, and a different formula for each shape of the test as in [19]), aperture size, mixotrophy, aperture position, test compression, and test material. Test length, test width, and aperture size were measured for ~ 5 replicates of each taxon in each sample. As some taxa were present in low abundance, not every recorded taxon was represented, but we aimed to cover at least 80% of the community by the measurements [26]. When five replicates were not feasible within reasonable time, we used the mean value calculated from the measurements from the same study site. Categorical traits were converted to binary format, assigning a value of 1 if a taxon exhibited the trait and 0 if it did not (see Supplementary Table 2). Finally, the community weighted mean (CWM) was calculated for each trait in each plot by quantifying the mean trait value of the taxon present in the community, weighted by their relative abundance [27]. 

To address the impact of selected environmental variables (Supplementary Table 3) on TA community composition and traits, we applied data from Kokkonen* et al*., (2019) [5] for soil nutrient concentrations and soil pH, and from Kuuri-Riutta *et al*., 2025 [21] for *Sphagnum* water content, WT (where negative values indicate water table below peat surface), soil temperature at the depths of 5 and 15 cm, and shading intensity (Fig. 1, Supplementary Fig. 1). To address the impact of different vegetation types on TA, we used vegetation inventory data recorded by Köster* et al*., (2023) [22]. All environmental data as well as vegetation coverages were recorded in the permanent sampling points, from which the testate amoeba samples were also collected.

### Statistical Analyses

The total TA count differed among samples (range 117–566), and the number of taxa found in a sample was correlated with the total count. Therefore, we omitted all taxa with relative abundance below the threshold of 0.8% (minimum relative abundance on the plot with the smallest number of observations) to achieve a dataset similar to rarefied data, yet without the random impact of rarefaction per se and assessed the community data as relative abundances thereafter. This resulted in a harmonized dataset that retained 52 of the 66 initially identified taxa (see Supplementary Table 4).

All analyses were conducted with R version 4.4.0 [28]. To analyze the differences in TA communities and traits between the sites and treatments, we used a non-metric multidimensional scaling (NMDS) from package vegan [29]. To assess the differences in TA communities among sites and treatments, we applied permutational multivariate ANOVA (*adonis*). The differences in the multivariate homogeneity of group dispersions were assessed with the function *betadisper*.

The alpha diversity of testate amoeba taxa was quantified as taxon richness, Simpson’s diversity index, and Hill’s evenness. Simpson’s diversity index was calculated using package vegan [29]. Taxon richness was recorded as the number of taxa in each sample. Hill’s evenness was calculated as Simpson’s index divided by taxon richness.

To test whether TA taxonomic composition and traits differ significantly among the sites and between the WLD and control treatments, multivariate analysis of variance (MANOVA) in package mvabund [30] was used, MANOVA was also used to recognize site and treatment preferences of TA taxa. We limited the inspection to the most common taxa with an abundance of > 10% at least in one sample. We used two-way-ANOVA and Tukey’s *post hoc* test to assess how traits responded to WLD within each of the three sites. When needed, data was square root or cubic root transformed to meet the parametric assumptions.

The optimal water table depth and tolerance range were obtained for each taxon by building a transfer function in package rioja [31]. We used the harmonized relative abundances and WT measurements repeated every second week over the summer 2022 as a training set. We used weighted averaging (WA) as it is rather robust to spatial autocorrelation and, therefore, valid for clustered datasets [32].

To assess how the most abundant taxa and traits reflected environmental variables and prevailing plant groups and to illustrate correlations between these variables, we run a fourth-corner analysis in the package corrplot [33].

## Results

### Testate Amoeba Diversity in the Six Study Areas

Site fertility had a positive main impact on taxon richness (p-value 0.017), Simpson’s diversity index (p-value 0.0003), and evenness (p-value 0.006), while the WLD treatment had a negative main impact only on Simpson’s index (p-value 0.03). According to pairwise comparisons (Tukey), taxon richness was significantly higher in the rich fen control area than the bog control area, Simpson’s index was higher in the rich fen control and WLD, and in the poor fen control area compared to the poor fen WLD and bog, and evenness was higher in the poor fen control compared to the bog control (Supplementary Fig. 2).

There were 20 taxa present in the fens but absent from the bog (see Supplementary Table 4), but only one taxon, *Pseudodifflugia fascicularis*, appeared exclusively in the bog.

### Testate Amoeba Community and Trait Composition in the Three Sites

**Fig. 2:**
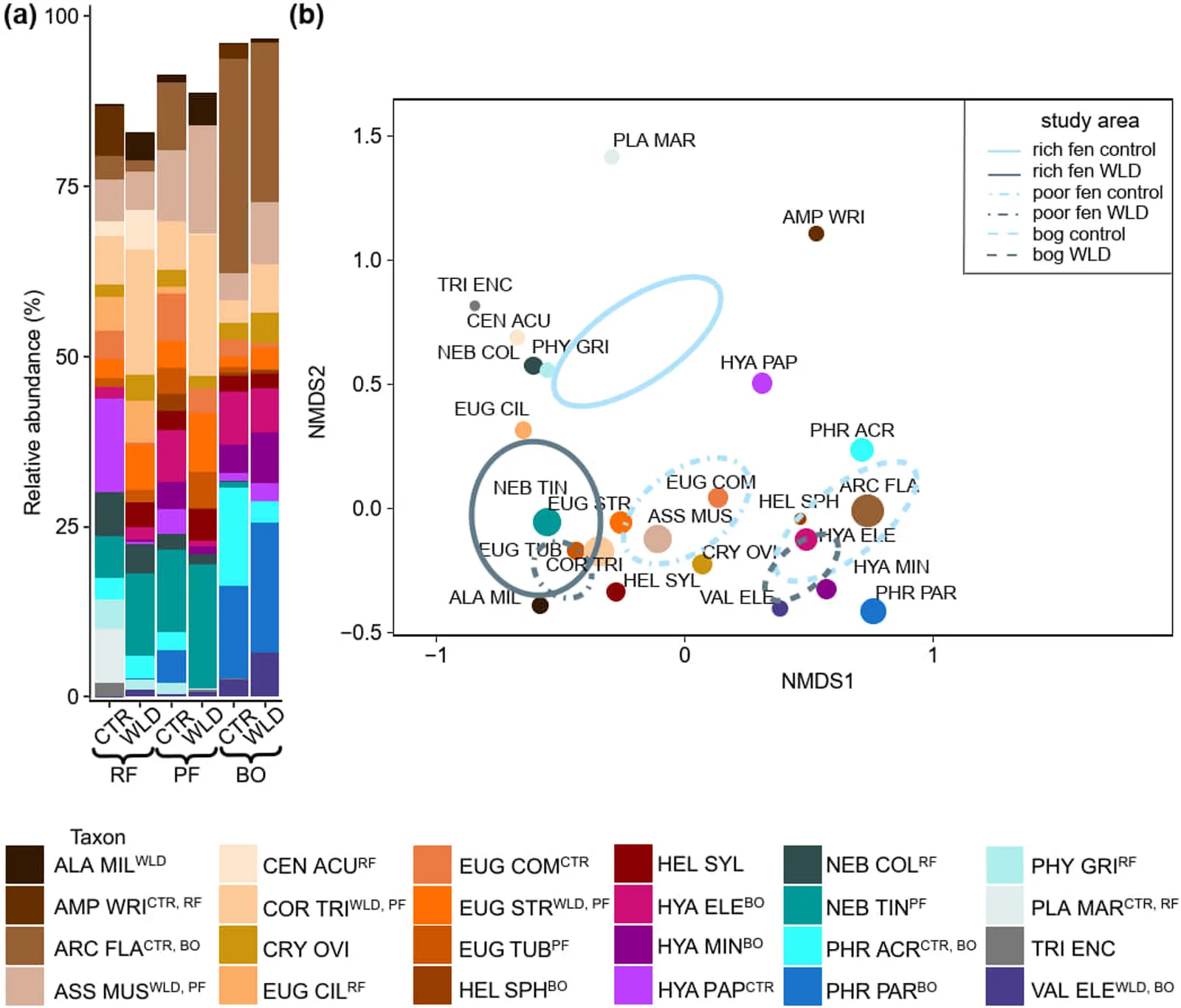
Overview of the most common taxa in the TA community across the WLD experiment in the three peatland types (**a**) and their location in the NMDS ordination (**b**). Here we only show selected taxa with relative abundance > 10% in at least one plot. See the abbreviations of taxon names in Supplementary Table 6. The superscript after the taxon name shows if the taxon indicates control conditions or WLD treatment (CTR = Control, WLD = Water level drawdown) or certain peatland type (RF = Rich fen, PF = Poor fen, BO = Bog). The size of the dots is scaled according to the relative abundance. The fit of all the presented environmental variables had a p-value of 0.001, except for SoilT5, that had a p-value of 0.003

Site was the strongest predictor of both community and trait composition (Supplementary Table 5). In the rich fen, the dominant taxa in the control area were *Hyalosphenia papilio* (average relative abundance 14%), *Planocarina marginata* (8%), and *Amphitrema wrightianum* (7%), and in the WLD area *Corythion-Trinema* type (18%), *Nebela tincta* type (12%), and *Euglypha strigosa* (7%) (Fig. 2, Supplementary Table 4). *Centropyxis acuelata*, *Euglypha ciliata*,* Nebela collaris*, and *Physochila griseola* were less abundant but preferred by nutrient-rich conditions (Supplementary Table 5). The poor fen control area was characterized by *N. tincta* type (12%), *Assulina muscorum* (10%), and *Archerella flavum* (10%), and the poor fen WLD area by *Corythion-Trinema* type (21%), *N. tincta* type (18%), and *A. muscorum* (16%). In both bog control and bog WLD areas, the most common taxa were *A. flavum* (32% and 23% in the control and WLD, respectively) and *Phryganella* spp. (28% and 22% in the control and WLD area, respectively).

**Fig. 3:**
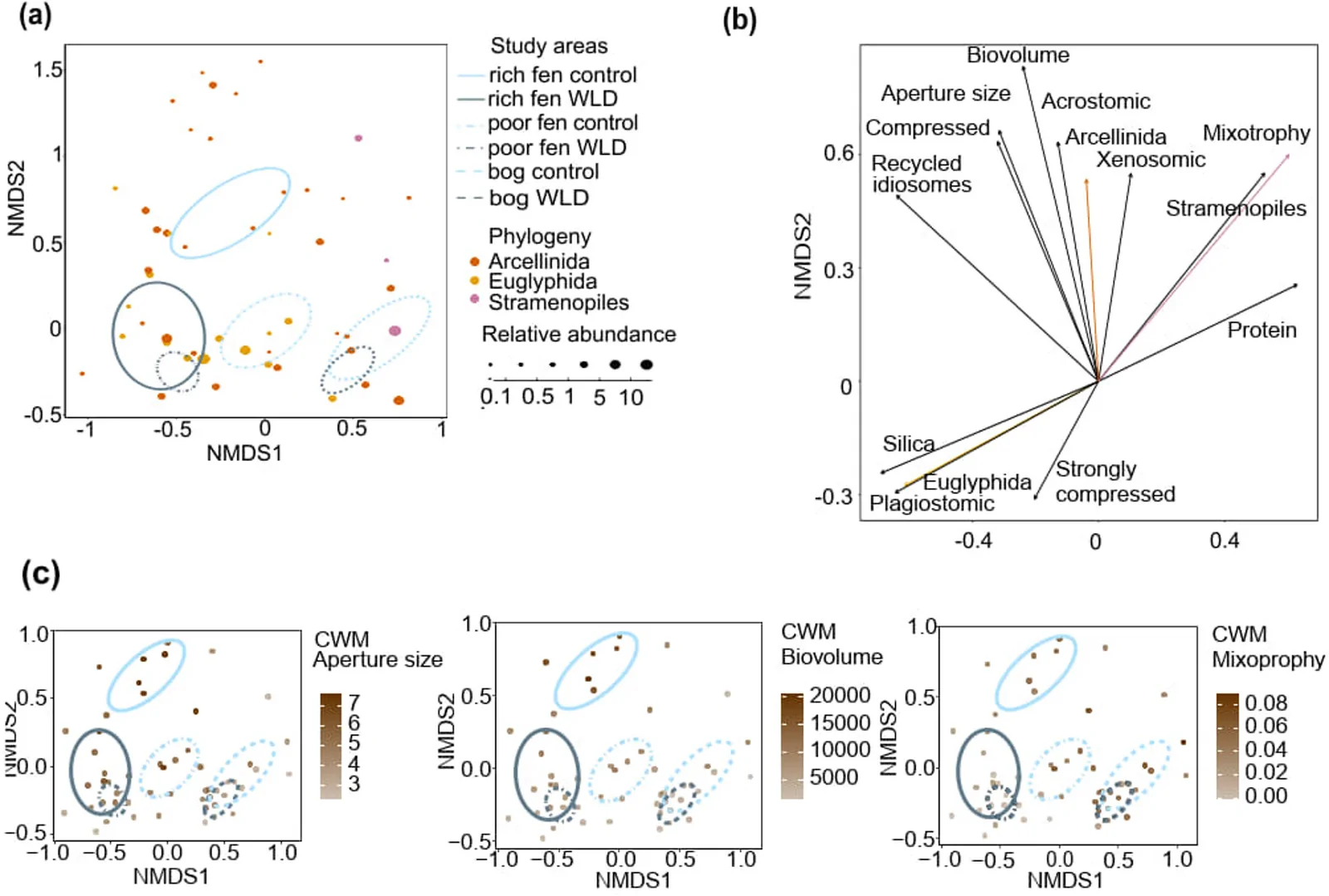
**(a)** NMDS where each species is coloured according to phylogeny. Traits as arrows in NMDS ordination. **(c)** NMDS where the study points are colored according to community weighted mean value within the range. See *envfit* statistics in Supplementary Table 7

The community weighted mean of biovolume, aperture size, recycled idiosomes and silica as test material, acrostomic and plagiostomic apertures, and compressed tests decreased along the nutrient gradient (Fig. 3). The CWM of strongly compressed tests and siliceous-organic as test material peaked in the poor fen.

### Differences in Testate Amoeba Community Composition between Water Level Treatments

**Fig. 4:**
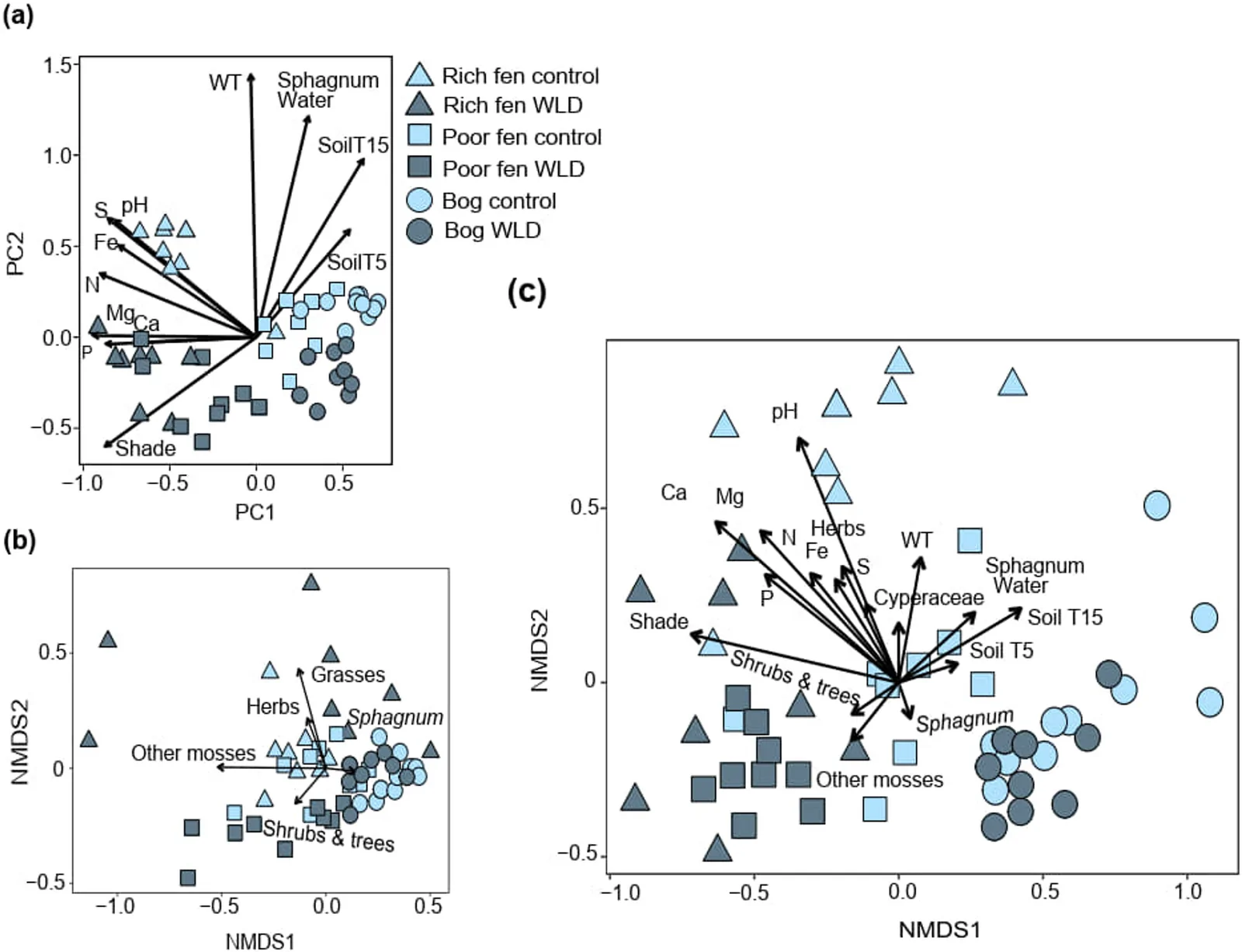
The study plots in **(a)** PCA ordination based on environmental variables, **(b)** NMDS ordination based on vegetation, and **(c)** NMDS ordination based on TA taxa, where the fit of the same environmental variables and vegetation groups are shown as vector arrows (see Supplementary Table 3 for the description of environmental variables and Supplementary Table 8 for *envfit* results). In the NMDS of the community, all sites differed from each other (*adonis*, p-value = 0.001) and the treatment impact was alike highly significant (p-value = 0.001). The variation within study areas (*betadispersion*) was not affected by the WLD treatments (p-value = 0.46), but it was higher in the rich fen compared to the other two sites (p-value < 0.001)

Abiotic variables, vegetation, and TA community composition all showed the largest differences between treatments in the rich fen and the smallest in the bog (Fig. 4). While TA communities differed from each other among the control areas, the communities in the rich fen and poor fen WLD areas were clustered together in NMDS (Fig. 4). A similar pattern was observed in the PCA based on abiotic characteristics, but not in the NMDS based on the vegetation (Fig. 4). Abiotic variables were stronger predictors of TA community composition than vegetation type (Supplementary Table 8).

Altogether 11 taxa showed site-independent preference for either WLD or undrained (control) conditions: *Alabasta militaris*, *A. muscorum*, *E. strigosa*, *Valkanovia elegans,* and *Corythion-Trinema* type preferred WLD, while *A. wrightianum*, *A. flavum*,* Euglypha compressa*,* H. papilio*,* P. marginata*, and *Phryganella acropodia* preferred undrained conditions (Supplementary Table 5). There was also site-treatment interaction: differences in the abundances of *A. flavum* and *H. papilio* between treatments were larger in the fen sites compared to the bog, while those in the abundance of *P. acropodia* were stronger in the bog. In addition, *Heleopera sylvatica* and *N. tincta* type preferred WLD and *Hyalosphenia elegans* and *Trinema enchelys* undrained conditions only in the fens. Cf. *P. paradoxa* and *Hyalosphenia minuta* preferred WLD in the bog, but the undrained control area in the poor fen.

### Differences in Testate Amoeba Functional Traits between Water Level Treatments

**Fig. 5:**
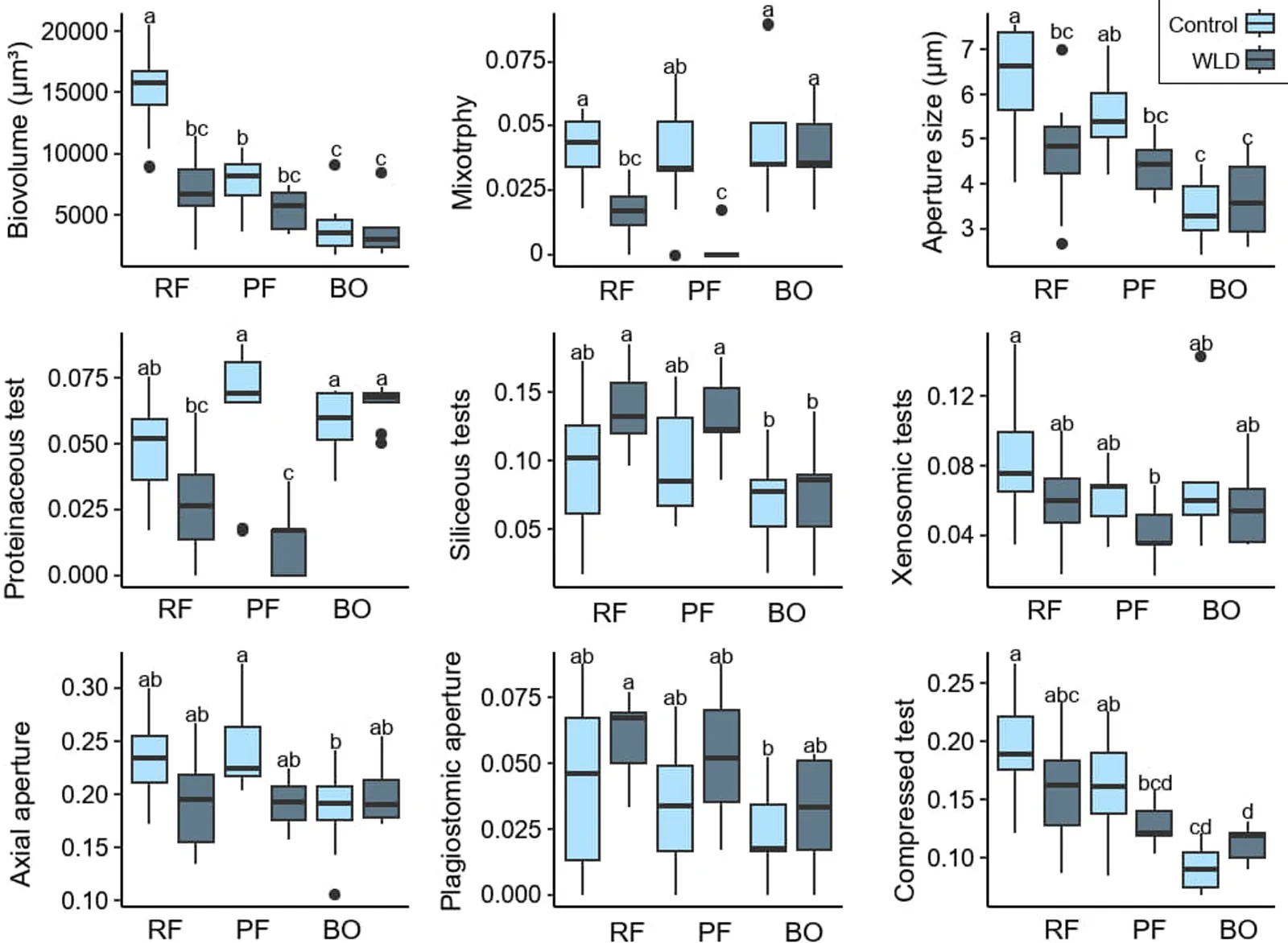
Functional traits significantly impacted by the water level drawdown treatment or the site*treatment interaction (MANOVA p value < 0.05). The letters indicate significant differences in the pairwise comparison. RF = Rich fen, PF = Poor fen, BO = Bog

The difference in traits between the treatments was the largest in the rich fen and the smallest in the bog (Supplementary Table 5, Figs. 3 and 5). While MANOVA showed a significant difference between treatments as a main effect for several traits, these were mostly driven by the responses in the fens and displayed rather modest changes in the bog (Supplementary Table 9). The WLD areas were characterized by relatively small biovolume and apertures, siliceous tests (on average, 35% of the taxa in the WLD areas), and plagiostomic apertures (on average, 16% of the taxa in the WLD areas). Traits more common in the control areas than the WLD areas included mixotrophy (on average, 26% of the taxa in the control areas), proteinaceous tests (on average, 30% of the taxa in the control areas), xenosomic tests (on average, 25% of the taxa in the control areas), and axial apertures (on average, 84% of the taxa in the control areas). (Fig. 5, Supplementary Table 5). In the WLD areas, order Euglyphida was more common and Stramenopiles less common than in the control areas. Arcellinida was less abundant in the fen WLD areas compared to the controls (Supplementary Fig. 3).

### Environmental Variables Driving TA Community and Trait Composition

**Fig. 6:**
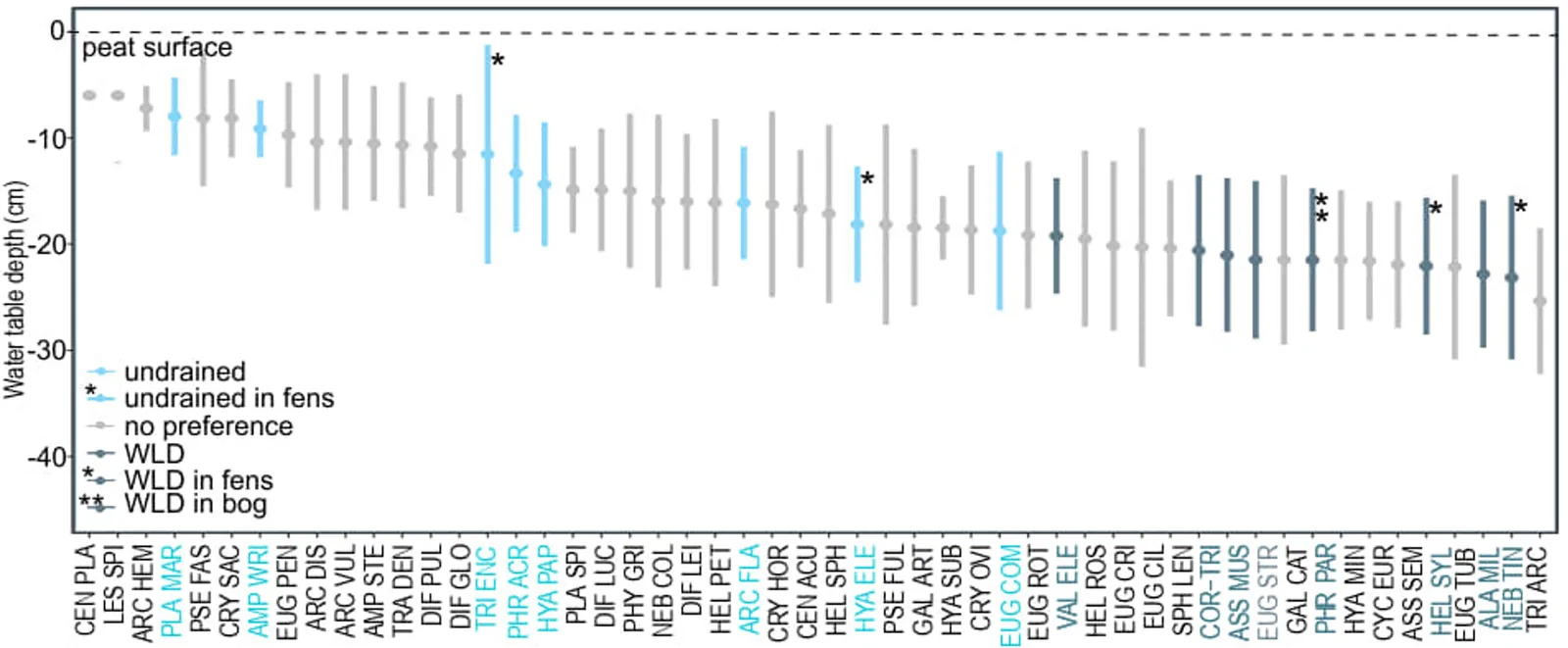
Water table depth optima and tolerance ranges. Taxa that preferred undrained areas are highlighted on light blue and taxa that preferred WLD areas are highlighted in dark blue. See the abbreviations of taxon names in Supplementary Table 6

Optimal water table depths ranged from − 19 cm to − 23 cm for WLD-favoring and from − 8 cm to − 18 cm for undrained-favoring taxa (Fig. 6). While the tolerance ranges were partly overlapping for the proxies for WLD and undrained conditions, WLD proxies preferred deeper water tables than the proxies for undrained conditions.

**Fig. 7:**
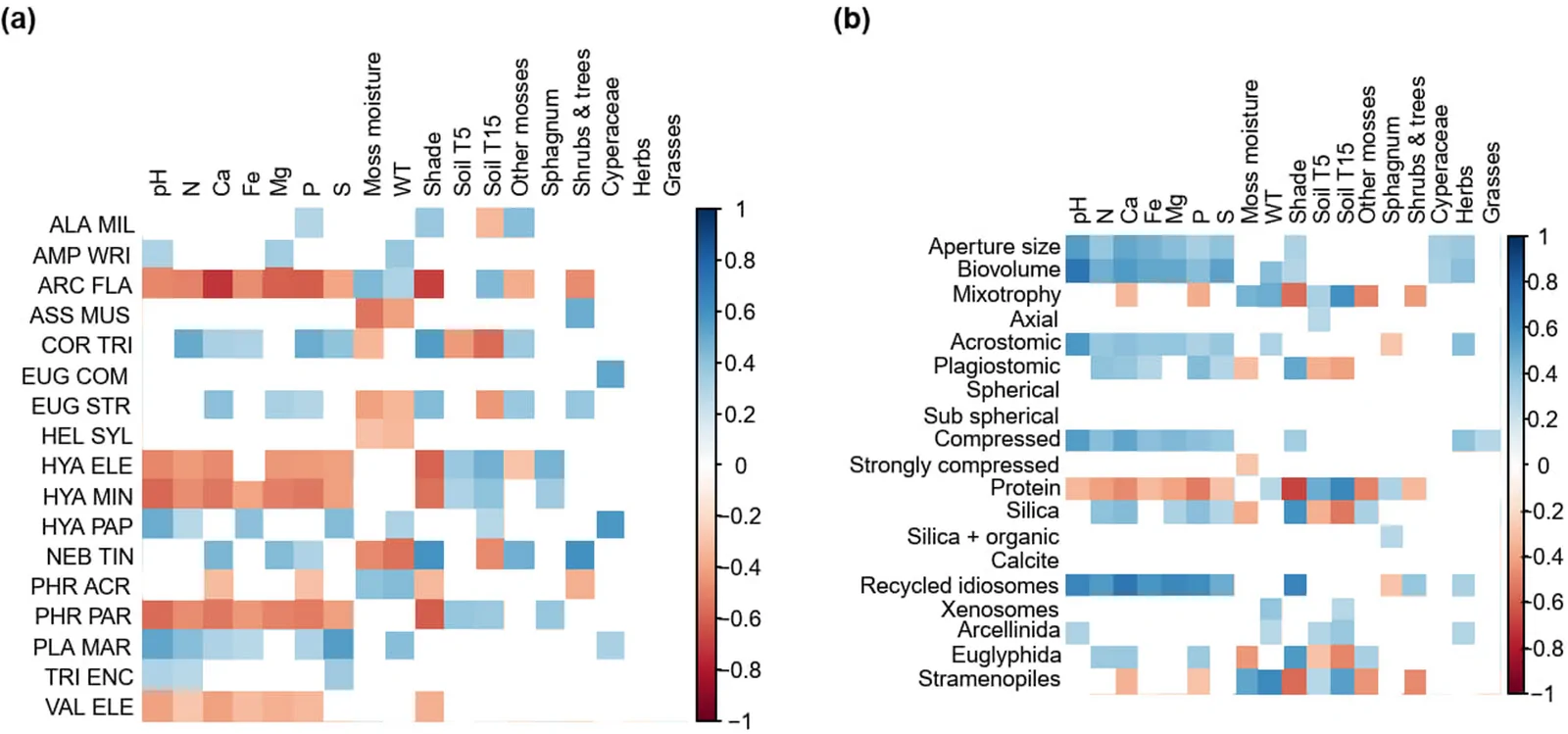
Fourth corner analysis illustrating correlations between environmental variables and (a) selected TA taxa, and (b) TA traits

TA taxa and traits were affected mostly by abiotic variables and less by plant types. Most WLD- or undrained-preferring taxa correlated with WT or *Sphagnum* moisture content (Fig. 7, see site-wise correlations in Supplementary Figs. 4–5), but many taxa and traits were equally impacted by pH, nutrient concentrations, and shading.

## Discussion

We quantified the differences in testate amoeba communities and traits between undrained boreal peatlands and their corresponding counterparts that have experienced decadal-scale, moderate drying and associated habitat changes (originally similar based on their vegetation and measured water table depths). The differences in both the composition and traits of testate amoeba between the treatments were regulated by site fertility and matched the documented changes in vegetation and abiotic characteristics [5, 21]. Our results imply that TA might be a strong proxy for the sensitivity or resistance of peatland ecosystems to environmental pressure and, especially, ongoing climate change.

### The Impacts of Site Fertility on Testate Amoeba Communities, Diversity, and Functional Traits

Our results show that TA communities and functional traits reflect differences in abiotic conditions and, to a lesser extent, plant groups along the site fertility gradient in boreal peatlands, in line with [34–37]. By monitoring TA across three peatland types we show that plagiostomic apertures and compressed tests, which are associated with dryness [19, 38, 39], were more common in the fens than in the bog, correlating directly with nutrient concentrations. Proteinaceous tests, on the other hand, were the most common in the bog, agreeing with [20]. Furthermore, fens displayed larger tests and apertures compared to the bog, and these traits were positively correlated to pH, nutrient concentrations, and fen-typical vegetation - Cyperaceae and herbs. This finding supports those of some previous studies [19, 39] but contrasts others, where small taxa replaced large taxa along a poor fen-rich fen gradient [40] and as a response to lake eutrophication [41]. Agreeing with existing literature [e.g., 11], biovolume also benefited from near-surface WT, and the rich fen was wetter than the other sites. These results support that site fertility may be considered the main ecological gradient in peatlands to which all biota and functions respond [42]. In addition, large biovolume and aperture size have been previously associated with a high trophic position [43, and references therein]. Therefore, it is possible that differences in food sources partly explain the differences observed across peatland types.

Fen TA communities were more diverse than bog communities, agreeing with existing literature that explains this by both abiotic and vegetation gradients, especially brown mosses hosting more diverse TA communities than *Sphagnum* mosses [12, 35, 40, 44]. Here, prevailing plant groups did not explain the composition of TA communities as strongly as abiotic variables. One limitation in our dataset is that we sampled only the dominant mosses, that was *Sphagnum* spp. except for two study points dominated by *Dicranum polysetum*. In addition, some taxa (e.g., *Difflugia* spp., *Cryptodifflugia oviformis*) occupy deeper sections of *Sphagnum* mosses than what was sampled for this study, i.e., below 3 cm. Thus, these taxa may be underrepresented, and some TA diversity as well as some relevant indicator taxa may have been ignored in this dataset.

### The Differences in Testate Amoeba Communities and Diversity between Treatments

Supporting our hypothesis, TA communities differed between the WLD and control areas most notably in the rich fen and least in the bog. A similar pattern has been observed for abiotic environmental variables and vegetation [5]. In line with previous research [45], this indicates that bog TA communities are more resistant to decadal-scale drying.

As stated before [e.g., 12, 36], site fertility-related differences in the response of TA communities should be accounted for when applying TA to reconstruct WT as a single variable. While ombrotrophic bogs receive all water and nutrients from the atmosphere, the ecohydrology of fens is more complex with several intercorrelated environmental gradients [42]. Therefore, in bogs, WT tends to be the strongest environmental variable controlling TA community composition [7]. Moreover, boreal ombrotrophic bogs are so nutrient-poor that changes in WT are rarely accompanied by establishment of a tree stand [46]. This makes the interpretation of paleo assemblages straightforward and has allowed the use of TA as a semi-quantitative WT proxy [e.g., 7]. On the contrary, fens readily respond to drying by arboreal vegetation succession and associated changes in pH, nutrient concentrations, and shading [5], which was shown to affect TA communities in this study and previously [e.g., 20]. This indicates that in fens, TA reflect the shift in the ecosystem state rather than merely WT depth. This complicates the attempts to reconstruct WT as a single variable in fens or through fen-bog transition: the secondary changes in fens may either amplify the drying signal or cause false signals to WT reconstructions, while in bogs, *Sphagnum* raising capillarity water [47] may hinder drying signals. Thus, we recommend multiproxy approach, e.g., plant macrofossils combined with TA.

11 taxa had consistent treatment preferences across sites. Agreeing with existing literature, *P. marginata*, *A. wrightianum*,* P. acropodia*, *E. compressa*,* A. flavum*, and *H. papilio* preferred undrained conditions, while *Corythion-Trinema*-type, *A. militaris*, *E. strigosa*, *A. muscorum*, and *V. elegans* preferred WLD areas [7, 11, 12, 48, 49]. *H. elegans*,* H. sylvatica*, cf. *P. paradoxa*, and *H. minuta* had site-specific treatment preferences even though they were present in all sites. WT tolerance ranges were partly overlapping between taxa preferring undrained and WLD conditions. Many were also recorded from their non-preferred study areas. Thus, it is important to inspect community-level changes rather than the presence/absence of individual taxa and note that training sets collected from bogs might not be reliable when applied to fen profiles [9]. Here, the interpretation of climate-induced changes in TA community composition and traits is based on the comparison of undrained and experimentally drained study areas at one point of time. To gain more detailed information about the succession of TA communities after WLD or other disturbances, we recommend designing experiments that follow the succession of TA communities across time in future studies.

### The Differences in Testate Amoeba Functional Traits between Treatments

Supporting our hypothesis, TA traits differed between the WLD and control areas most notably in the rich fen and least in the bog. In the bog, no significant differences between the control and WLD areas were observed in the studied traits. Thus, our result suggests that the usefulness of TA traits as bioindicators of drying in ombrotrophic bogs may be limited, at least within the range of WT changes applied in this experiment. The finding is somewhat surprising, since no strong dependency on the site type in WLD responses of TA traits has been reported before, and a trait-based transfer function has been successfully developed for ombrotrophic peatlands [50]. A possible explanation is that the WT difference between the control and the WLD area was smaller in the bog than in the fens in the sampling year. Moreover, the bog hosted smaller, i.e., better drought-adapted [11, 16, 43] and references therein, taxa compared to fens. Thus, it seems likely that bog TA communities are adapted to persist in moderately drying conditions.

In the fens, communities in the WLD areas were characterized by smaller tests with smaller, plagiostomic apertures, siliceous tests, and notably fewer mixotrophs compared to the control areas. Small size and small, plagiostomic apertures are considered adaptations to drying, enabling movement in a thin water film and protecting against desiccation [43] and references therein]. Mixotrophs – especially *A. flavum* and *H. papilio* – suffered from deep WT, low *Sphagnum* water content, high shading intensity, and shrubs and trees, agreeing with previous studies [11, 20, 49]. The smaller proportion of proteinaceous test material is likely associated with the small number of mixotrophs, while the smaller proportion of xenosomic tests may be connected to the limited diversity and availability of mineral particles [50]. Many of the traits favored by WLD are manifested in the order Euglyphida, whose share was higher in the fen WLD areas compared to the control areas, while the opposite was observed in Arcellinida, agreeing with previous research [e.g., 50]. Overall, the results support previous studies that suggested that TA traits respond not only to water level but also to other environmental variables, in this case, to the increased tree coverage and associated abiotic changes in the fen WLD areas [16, 20, 38].

## Supplementary Information

Below is the link to the electronic supplementary material.

Supplementary Material 1 (DOCX 379 KB)

## Data Availability

The data that support the findings of this study is openly available in Zenodo research data storage service (10.5281/zenodo.17940365). The environmental data from Kokkonen *et al*., (2019) are stored in the Pangaea Data Library (doi: 10.1594/pangaea.904256), and those from Kuuri-Riutta *et al*., (2025) are stored in IDA research data storage service (10.23729/fd-bad048dd-eda3-380f-915a-580ccd150ec2). Vegetation data from Köster *et al*., (2023) are stored in the Dryad repository (doi: 10.5061/dryad.dz08kps39).
