# Supplementary material for: Assessing the Value of Testate Amoebae and their Functional Traits in Detecting Climate Change-Induced Peatland Drying
Source: Microb Ecol. 2025 Dec 20;89(1):35. doi: 10.1007/s00248-025-02682-2 (PMC12819454; doi:10.1007/s00248-025-02682-2)
Supplement: Supplementary file 1 — Supplementary Material 1 (DOCX 379 KB) [file 248_2025_2682_MOESM1_ESM.docx]

**Supplementary Information**

**Assessing the value of testate amoebae and their functional traits in detecting climate change- induced peatland drying**

Microbial Ecology

Olivia Kuuri-Riutta, Brunella Palacios Ganoza, Henni Ylänne, Edward A. D. Mitchell, Minna M. Väliranta, Eeva-Stiina Tuittila.

Corresponding author: Olivia Kuuri-Riutta, ^1^School of Forest Sciences, University of Eastern Finland, Joensuu, Finland, [olivia.kuuri-riutta@uef.fi](mailto:olivia.kuuri-riutta@uef.fi)

Supplementary Table 1. Dominant mosses in each sampling plot. *Sphagnum recurvum* complex refers to *Sphagnum angustifolium*, *Sphagnum flexuosum*, *and Sphagnum fallax*, and *Sphagnum magellanicum* complex refers to *Sphagnum medium* and *Sphagnum divinum*.

| Sampling plot | Site | Treatment | Dominant mosses |
| --- | --- | --- | --- |
| MEC1 | rich fen | control | *Sphagnum recurvum* complex |
| MEC2 | rich fen | control | *Sphagnum recurvum* complex |
| MEC3 | rich fen | control | *Sphagnum recurvum* complex*, Sphagnum magellanicum* complex |
| MEC4 | rich fen | control | *Sphagnum recurvum* complex |
| MEC5 | rich fen | control | *Sphagnum recurvum* complex |
| MEC6 | rich fen | control | *Sphagnum recurvum* complex |
| MEC7 | rich fen | control | *Sphagnum teres* |
| MEC8 | rich fen | control | *Sphagnum recurvum* complex |
| MED1 | rich fen | WLD | *Sphagnum capillifolium* |
| MED2 | rich fen | WLD | *Sphagnum recurvum* complex, *Sphagnum teres* |
| MED3 | rich fen | WLD | *Sphagnum capillifolium* |
| MED4 | rich fen | WLD | *Dicranum polysetum* |
| MED5 | rich fen | WLD | *Dicranum polysetum* |
| MED6 | rich fen | WLD | *Sphagnum russowii* |
| MED7 | rich fen | WLD | *Sphagnum recurvum* complex |
| MED8 | rich fen | WLD | *Sphagnum teres* |
| OLC1 | poor fen | control | *Sphagnum recurvum* complex, *Sphagnum papillosum* |
| OLC2 | poor fen | control | *Sphagnum papillosum, Sphagnum recurvum complex* |
| OLC3 | poor fen | control | *Sphagnum papillosum, Sphagnum recurvum complex* |
| OLC4 | poor fen | control | *Sphagnum papillosum, Sphagnum recurvum complex* |
| OLC5 | poor fen | control | *Sphagnum recurvum* complex |
| OLC6 | poor fen | control | *Sphagnum recurvum* complex |
| OLC7 | poor fen | control | *Sphagnum recurvum* complex, *Sphagnum papillosum* |
| OLC8 | poor fen | control | *Sphagnum papillosum, Sphagnum recurvum* complex |
| OLC9 | poor fen | control | *Sphagnum papillosum, Sphagnum recurvum* complex |
| OLD1 | poor fen | WLD | *Sphagnum recurvum* complex*, Sphagnum papillosum* |
| OLD2 | poor fen | WLD | *Sphagnum recurvum* complex, *Sphagnum papillosum* |
| OLD3 | poor fen | WLD | *Sphagnum recurvum* complex |
| OLD4 | poor fen | WLD | *Sphagnum recurvum* complex |
| OLD5 | poor fen | WLD | *Sphagnum recurvum* complex |
| OLD6 | poor fen | WLD | *Sphagnum recurvum* complex*, Sphagnum magellanicum* complex |
| OLD7 | poor fen | WLD | *Sphagnum recurvum* complex |
| OLD8 | poor fen | WLD | *Sphagnum recurvum* complex |
| OLD9 | poor fen | WLD | *Sphagnum recurvum* complex |
| OMC1 | bog | control | *Sphagnum fuscum, Sphagnum rubellum* |
| OMC2 | bog | control | *Sphagnum cuspidatum* |
| OMC3 | bog | control | *Sphagnum cuspidatum* |
| OMC4 | bog | control | *Sphagnum majus, Sphagnum fuscum* |
| OMC5 | bog | control | *Sphagnum fuscum, Sphagnum majus* |
| OMC6 | bog | control | *Sphagnum rubellum* |
| OMC7 | bog | control | *Sphagnum balticum, Sphagnum rubellum* |
| OMC8 | bog | control | *Sphagnum fuscum* |
| OMC9 | bog | control | *Sphagnum fuscum, Sphagnum rubellum* |
| OMC10 | bog | control | *Sphagnum fuscum, Sphagnum rubellum* |
| OMD1 | bog | WLD | *Sphagnum balticum* |
| OMD2 | bog | WLD | *Sphagnum balticum, Sphagnum fuscum* |
| OMD3 | bog | WLD | *Sphagnum fuscum* |
| OMD4 | bog | WLD | *Sphagnum rubellum, Sphagnum balticum* |
| OMD5 | bog | WLD | *Sphagnum fuscum* |
| OMD6 | bog | WLD | *Sphagnum fuscum, Sphagnum balticum* |
| OMD7 | bog | WLD | *Sphagnum fuscum* |
| OMD8 | bog | WLD | *Sphagnum fuscum* |
| OMD9 | bog | WLD | *Sphagnum balticum, Sphagnum rubellum* |

Supplementary Table 2. Functional traits of each taxon. Mixotrophy: 0 = heterotroph, 1 = mixotroph. Aperture position: 1 = axial, 2 = acrostomic, 3 = plagiostomic. Test compression: 1=spherical, 2=sub-spherical, 3=compressed, 4=strongly compressed . Test material: 1 = protein, 2 = silica, 3 = silica + organic, 4 = calcite, 5 = recycled idiosomes, 6 = xenosomes . Phylogeny: 1 = Arcellinida, 2 = Euglyphida, 3 = Stramenopiles.

| Taxon | Aperture size (µm) | Biovolume (µm³) | Mixotrophy | Aperture position | Test Compression | Test Material | Phylogeny |
| --- | --- | --- | --- | --- | --- | --- | --- |
| *Alabasta militaris* | 17 | 10495 | 0 | 1 | 3 | 5 | 1 |
| *Amphitrema stenostoma* | 10 | 21328 | 1 | 1 | 3 | 6 | 3 |
| *Amphitrema wrightianum* | 10 | 39665 | 1 | 1 | 3 | 6 | 3 |
| *Arcella hemispherica* | 15 | 6619 | 0 | 2 | 4 | 1 | 1 |
| *Arcella vulgaris* | 27 | 31161 | 0 | 2 | 3 | 1 | 1 |
| *Archerella flavum* | 7 | 6521 | 1 | 1 | 3 | 1 | 3 |
| *Assulina muscorum* | 13 | 6714 | 0 | 1 | 4 | 3 | 2 |
| *Assulina seminulum* | 21 | 29515 | 0 | 1 | 4 | 3 | 2 |
| *Centropyxis aculeata* | 26 | 165856 | 0 | 2 | 3 | 6 | 1 |
| *Centropyxis platystoma* | 34 | 68669 | 0 | 2 | 3 | 6 | 1 |
| *Corythion constricta* | 11 | 5906 | 0 | 3 | 4 | 2 | 2 |
| *Corythion dubium* | 10 | 2526 | 0 | 3 | 4 | 2 | 2 |
| *Cryptodifflugia horrida* type | 10 | 723 | 0 | 1 | 2 | 4 | 1 |
| *Cryptodifflugia oviformis* | 5 | 517 | 0 | 1 | 2 | 4 | 1 |
| *Cryptodifflugia sacculus* | 10 | 1224 | 0 | 1 | 2 | 6 | 1 |
| *Cyclopyxis eurystoma* | 33 | 1398 | 0 | 2 | 1 | 6 | 1 |
| *Difflugia globulosa* | 46 | 248283 | 0 | 1 | 1 | 6 | 1 |
| *Difflugia leidyi* | 37 | 115377 | 0 | 1 | 3 | 6 | 1 |
| *Difflugia lucida type* | 15 | 17455 | 0 | 1 | 3 | 6 | 1 |
| *Difflugia pulex* | 11 | 5591 | 0 | 1 | 2 | 6 | 1 |
| *Euglypha ciliata* | 19 | 17076 | 0 | 1 | 3 | 2 | 2 |
| *Euglypha compressa* | 25 | 32642 | 0 | 1 | 4 | 2 | 2 |
| *Euglypha cristata* | 7 | 2152 | 0 | 1 | 2 | 2 | 2 |
| *Euglypha rotunda* | 8 | 1547 | 0 | 1 | 3 | 2 | 2 |
| *Euglypha strigosa* | 21 | 16561 | 0 | 1 | 3 | 2 | 2 |
| *Euglypha tuberculata* | 14 | 13347 | 0 | 1 | 2 | 2 | 2 |
| *Galeripora artocrea* | 21 | 49456 | 0 | 2 | 3 | 1 | 1 |
| *Galeripora catinus* | 22 | 37176 | 0 | 2 | 3 | 1 | 1 |
| *Galeripora discoides* | 46 | 34361 | 0 | 2 | 4 | 1 | 1 |
| *Heleopera petricola* | 38 | 68024 | 0 | 1 | 3 | 6 | 1 |
| *Heleopera rosea* | 33 | 55688 | 0 | 1 | 3 | 6 | 1 |
| *Heleopera sphagni* | 38 | 75432 | 1 | 1 | 3 | 6 | 1 |
| *Heleopera sylvatica* | 24 | 25025 | 0 | 1 | 3 | 6 | 1 |
| *Hyalosphenia elegans* | 18 | 24312 | 0 | 1 | 3 | 1 | 1 |
| *Hyalosphenia minuta* | 9 | 2039 | 0 | 1 | 3 | 1 | 1 |
| *Hyalosphenia papilio* | 33 | 69770 | 1 | 1 | 3 | 1 | 1 |
| *Hyalosphenia subflava* | 17 | 18267 | 0 | 1 | 3 | 5 | 1 |
| *Nebela collaris* | 29 | 103342 | 0 | 1 | 3 | 5 | 1 |
| *Nebela tincta* type | 20 | 51746 | 0 | 1 | 3 | 5 | 1 |
| *Phryganella acropodia* | 24 | 399 | 0 | 2 | 2 | 6 | 1 |
| cf. *Phryganella paradoxa* | 7 | 114 | 0 | 1 | 2 | 6 | 1 |
| *Physochila griseola* | 23 | 78551 | 0 | 1 | 3 | 6 | 1 |
| *Placocista spinosa* | 31 | 39407 | 1 | 1 | 4 | 2 | 0 |
| *Planocarina marginata* | 36 | 174605 | 0 | 1 | 3 | 5 | 1 |
| *Pseudodifflugia fulva* type | 6 | 3225 | 0 | 1 | 2 | 6 | 2 |
| *Sphenoderia lenta* | 11 | 2375 | 0 | 3 | 3 | 2 | 2 |
| *Tracheleuglypha dentata* | 9 | 9505 | 0 | 1 | 2 | 2 | 2 |
| *Trigonopyxis arcula* | 16 | 81190 | 0 | 2 | 2 | 6 | 1 |
| *Trinema complanatum* | 11 | 5948 | 0 | 3 | 3 | 2 | 2 |
| *Trinema enchelys* | 10 | 13406 | 0 | 3 | 3 | 2 | 2 |
| *Trinema lineare* | 6 | 1914 | 0 | 3 | 2 | 2 | 2 |
| *Valkanovia elegans* | 8 | 2806 | 0 | 1 | 2 | 2 | 2 |

Supplementary Table 3. The summary of the measured environmental variables and vegetation groups.

| **Variable** | **Abbreviation** | **Unit** | **Measurement year** | **Repetitions** | **Description or reference** |
| --- | --- | --- | --- | --- | --- |
| Peat nutrient concentration (Ca, Fe, N, P, Mg, S) |  | g^-1^ kg^-1^ | 2016 | 1 | Kokkonen *et al*. (2019) |
| Water table level | WT | cm | 2022 | Every 2^nd^ week,  Jun-Aug | A distance from peat surface to water level, measured from permanent water wells. Negative value means below peat surface. |
| pH |  |  | 2016 | 1 | Kokkonen *et al*. (2019) |
| Shading intensity | Shade | % of the    initial PAR | 2022 | 1 | Quantifies the shading intensity from vascular plant coverage. 100–(PAR_below_/PAR_above_),  where PAR_below_ is photosynthetically active radiation below the field layer and PAR_above_ is photosynthetically active radiation above field layer. |
| *Sphagnum*  water content | SphagnumWater | % of weigh | 2022 | 1 | 100–(*Sphagnum* dry mass / *Sphagnum* fresh mass) |
| Soil temperature at 5 and 15 cm depth | SoilT5, SoilT15 | °C | 2022 | Every 2^nd^ week  Jun-Aug | Measured from the permanent sampling plots. |
| Coverage of *Sphagnum* spp*.* | *Sphagnum* | % | 2021 | 1 | visually estimated proportional coverage on the sampling plot [1] |
| Coverage of non-Sphagnum mosses | Other mosses | % | 2021 | 1 | visually estimated proportional coverage on the sampling plot [1] |
| Coverage of sedge vegetation (i.e., Carex spp., Eriophorum spp., Tricophorum spp.) | Cyperaceae | % | 2021 | 1 | visually estimated proportional coverage on the sampling plot [1] |
| Coverage of shrubs and small trees growing at the ground layer. | Shrubs & trees | % | 2021 | 1 | visually estimated proportional coverage on the sampling plot [1] |
| Coverage of herbaceous plants (e.g., *Menyanthes trifoliata, Potentilla palustre*) | Herbs | % | 2021 | 1 | visually estimated proportional coverage on the sampling plot [1] |
| Coverage of grasses (i.e., *Agrostis* sp., *Calamagrostis* sp.) | Grasses | % | 2021 | 1 | visually estimated proportional coverage on the sampling plot [1] |


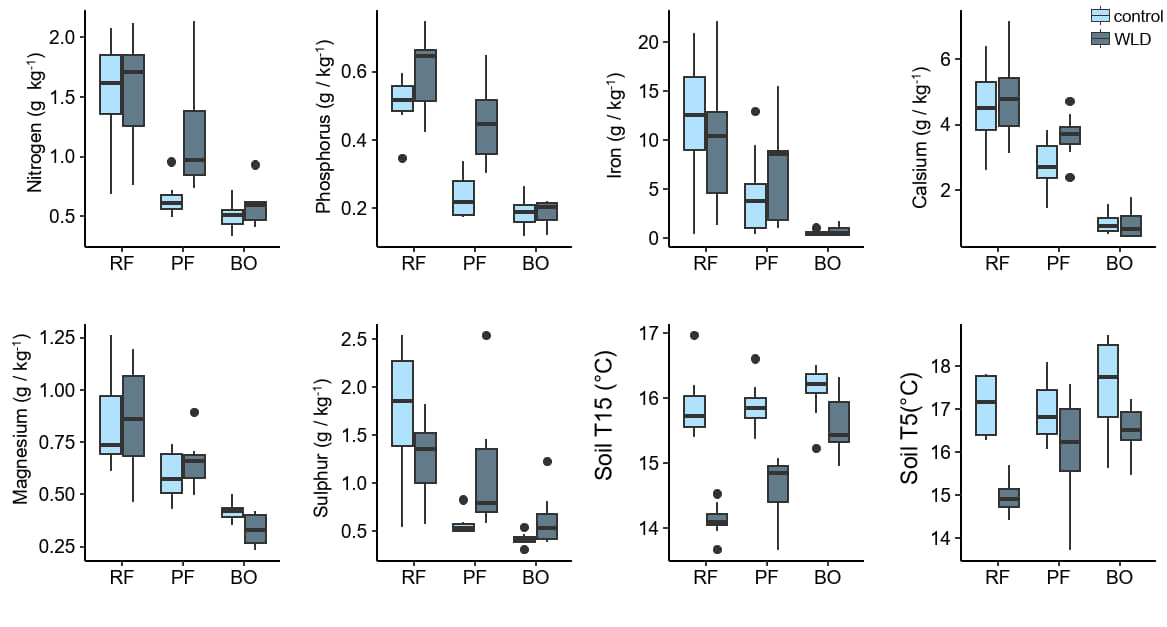


Supplementary Figure 1. Data on environmental variables in Lakkasuo experiment. SoilT = Soil temperature, WLD = water level drawdown. Nutrient data originally published in Kokkonen *et al*. (2019) [2] and soil temperature data in Kuuri-Riutta *et al*. (2025) [3].

Supplementary Table 4. Taxa included in the final dataset and the average relative abundance (%) of each taxa in each study area.

| Taxon | Rich fen control | Rich fen WLD | Poor fen control | Poor fen WLD | Bog control | Bog WLD |
| --- | --- | --- | --- | --- | --- | --- |
| *Alabasta militaris* | 0.5 | 4.3 | 1.1 | 4.9 | 0.2 | 0.6 |
| *Amphitrema stenostoma* | 0.4 | 0.0 | 0.0 | 0.0 | 0.4 | 0.2 |
| *Amphitrema wrightianum* | 7.3 | 0.0 | 0.0 | 0.0 | 2.3 | 0.2 |
| *Arcella hemispherica* | 0.5 | 0.0 | 0.0 | 0.0 | 0.0 | 0.0 |
| *Arcella vulgaris* | 0.6 | 0.0 | 0.0 | 0.0 | 0.0 | 0.0 |
| *Archerella flavum* | 3.5 | 1.6 | 9.9 | 0.0 | 31.5 | 23.3 |
| *Assulina muscorum* | 6.1 | 5.7 | 10.4 | 15.8 | 4.0 | 9.1 |
| *Assulina seminulum* | 0.4 | 0.0 | 3.2 | 2.9 | 0.7 | 1.3 |
| *Centropyxis acuelata* | 2.2 | 5.8 | 0.0 | 0.2 | 0.0 | 0.0 |
| *Centropyxis platystoma* | 1.1 | 0.0 | 0.0 | 0.0 | 0.0 | 0.0 |
| *Corythion-trinema* type | 7.1 | 18.3 | 7.2 | 20.8 | 3.3 | 7.3 |
| *Cryptodifflugia horrida* type | 0.0 | 0.2 | 0.2 | 0.0 | 0.2 | 0.0 |
| *Cryptodifflugia oviformis* | 1.8 | 4.0 | 2.5 | 1.7 | 2.4 | 4.4 |
| *Cryptodifflugia sacculus* | 0.2 | 0.0 | 0.0 | 0.0 | 0.5 | 0.0 |
| *Cyclopyxis eurystoma* | 0.0 | 2.2 | 0.0 | 0.8 | 0.5 | 0.4 |
| *Difflugia globulosa* | 0.9 | 0.0 | 0.0 | 0.0 | 0.2 | 0.2 |
| *Difflugia leidyi* | 0.2 | 0.0 | 0.0 | 0.0 | 0.0 | 0.0 |
| *Difflugia lucida* type | 1.1 | 0.0 | 0.0 | 0.0 | 0.0 | 0.0 |
| *Difflugia pulex* | 0.9 | 0.0 | 0.0 | 0.0 | 0.2 | 0.2 |
| *Euglypha ciliata* | 5.0 | 5.9 | 1.0 | 0.0 | 0.0 | 0.0 |
| *Euglypha compressa* | 4.1 | 0.4 | 7.1 | 3.7 | 2.6 | 0.8 |
| *Euglypha cristata* | 0.4 | 3.0 | 0.9 | 1.2 | 0.0 | 0.0 |
| *Euglypha penardi* | 0.2 | 0.0 | 0.0 | 0.0 | 0.2 | 0.0 |
| *Euglypha rotunda* | 0.9 | 4.4 | 0.4 | 1.9 | 0.4 | 0.6 |
| *Euglypha strigosa* | 2.9 | 6.7 | 3.9 | 8.7 | 1.4 | 3.1 |
| *Euglypha tuberculata* | 1.3 | 1.9 | 3.7 | 5.2 | 0.9 | 0.2 |
| *Galeripora artocrea* | 0.4 | 0.9 | 0.2 | 0.2 | 0.0 | 0.0 |
| *Galeripora catinus* | 0.2 | 0.8 | 0.8 | 0.6 | 0.0 | 0.0 |
| *Galeripora discoides* | 0.2 | 0.0 | 0.0 | 0.0 | 0.0 | 0.0 |
| *Heleopera petricola* | 0.4 | 0.0 | 0.9 | 0.2 | 0.0 | 0.0 |
| *Heleopera rosea* | 1.8 | 2.5 | 0.6 | 1.6 | 0.0 | 0.0 |
| *Heleopera sphagni* | 0.0 | 0.0 | 2.5 | 0.2 | 0.5 | 0.6 |
| *Heleopera sylvatica* | 0.0 | 3.7 | 2.8 | 4.6 | 2.3 | 2.1 |
| *Hyalosphenia elegans* | 1.5 | 1.7 | 7.7 | 0.8 | 7.7 | 6.5 |
| *Hyalosphenia minuta* | 0.0 | 0.4 | 3.8 | 1.2 | 4.2 | 7.5 |
| *Hyalosphenia papilio* | 13.9 | 0.4 | 3.8 | 0.0 | 1.2 | 2.7 |
| *Hyalosphenia subflava* | 0.0 | 0.2 | 0.0 | 0.0 | 0.0 | 0.2 |
| *Lesquereusia spiralis* | 0.2 | 0.0 | 0.0 | 0.0 | 0.0 | 0.0 |
| *Nebela collaris* | 6.4 | 4.3 | 2.3 | 1.5 | 0.0 | 0.0 |
| *Nebela tincta* type | 6.0 | 12.0 | 12.1 | 18.2 | 0.9 | 0.0 |
| *Phryganella arcopodia* | 3.2 | 3.3 | 2.7 | 0.0 | 14.6 | 3.2 |
| cf. *Phryganella paradoxa* | 0.0 | 0.2 | 4.9 | 0.0 | 13.6 | 18.9 |
| *Physochila griseola* | 4.4 | 1.5 | 1.5 | 0.0 | 0.0 | 0.0 |
| *Placiocista spinosa* | 0.4 | 0.9 | 0.9 | 0.0 | 0.0 | 0.2 |
| *Planocarina marginata* | 7.9 | 0.0 | 0.0 | 0.0 | 0.0 | 0.0 |
| *Pseudodifflugia fascicularis* | 0.0 | 0.0 | 0.0 | 0.0 | 0.2 | 0.0 |
| *Pseudodifflugia fulva* type | 0.0 | 0.4 | 0.2 | 0.6 | 0.5 | 0.0 |
| *Sphenoderia lenta* | 0.6 | 0.6 | 0.0 | 1.0 | 0.0 | 0.0 |
| *Tracheleuglypha dentata* | 0.7 | 0.4 | 0.2 | 0.0 | 0.0 | 0.0 |
| *Trigonopyxis arcula* | 0.0 | 0.4 | 0.0 | 0.4 | 0.0 | 0.0 |
| *Trinema enchelys* | 1.9 | 0.0 | 0.0 | 0.4 | 0.0 | 0.0 |
| *Valkanovia elegans* | 0.0 | 1.0 | 0.4 | 0.8 | 2.6 | 6.5 |


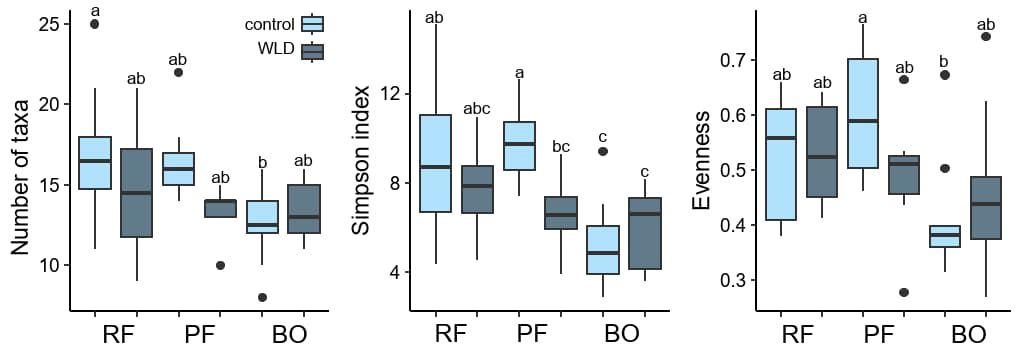


Supplementary Figure 2. Taxon richness, Simpson’s index, and Hill’s evenness of testate amoeba communities in the six study areas. The letters indicate significant differences in the pairwise comparison. RF = Rich fen, PF = Poor fen, BO = Bog.

Supplementary Table 5. Results of the multivariate generalized linear model (mvabund) on the impact of water level drawdown (treatment), site (rich fen, poor fen, and bog), and their interaction on the overall testate amoeba community structure and community weighted means of functional traits, and the univariate test results on the impact of water level drawdown (treatment), site (rich fen, poor fen, and bog), and their interaction on the individual TA taxa and functional traits. P-values refer to unadjusted p-values.

|  | **Treatment** | | **Site** | | **Site:Treatment** | | **Preference** |
| --- | --- | --- | --- | --- | --- | --- | --- |
|  | **dev** | **p** | **dev** | **p** | **dev** | **p** |  |
| Overall community | 125.91 | 0.001 | 376.99 | 0.001 | 168.12 | 0.001 |  |
| *Alabasta militaris* | 6.56 | 0.02 | 6.27 | 0.079 | 0.51 | 0.806 | WLD |
| *Amphitrema wrightianum* | 6.96 | 0.019 | 8.21 | 0.033 | 2.32 | 0.098 | CTR, RF |
| *Archerella flavum* | 11.62 | 0.001 | 23.26 | 0.001 | 27.62 | 0.001 | CTR, BO, more sensitive in fens |
| *Assulina muscorum* | 6.12 | 0.024 | 15.42 | 0.002 | 4.69 | 0.138 | WLD, PF |
| *Centropyxis acuelata* | 2.81 | 0.143 | 27.62 | 0.001 | 0.99 | 0.257 | RF |
| *Trinema enchelys* | 0.00 | 0.914 | 3.47 | 0.228 | 4.66 | 0.032 | CTR in RF (not present in bog) |
| *Cryptodifflugia oviformis* | 0.80 | 0.384 | 1.00 | 0.598 | 1.70 | 0.495 |  |
| *Euglypha ciliata* | 1.18 | 0.325 | 15.88 | 0.001 | 3.94 | 0.074 | RF |
| *Euglypha compressa* | 9.32 | 0.004 | 5.70 | 0.085 | 2.98 | 0.373 | CTR |
| *Euglypha strigosa* | 8.53 | 0.005 | 8.60 | 0.014 | 0.00 | 0.998 | WLD, PF |
| *Euglypha tuberculata* | 0.07 | 0.792 | 25.83 | 0.001 | 4.78 | 0.138 | PF |
| *Heleopera sphagni* | 2.33 | 0.189 | 11.84 | 0.013 | 3.60 | 0.115 | BO |
| *Heleopera sylvatica* | 4.63 | 0.062 | 2.70 | 0.313 | 15.95 | 0.003 | WLD in fens |
| *Hyalosphenia elegans* | 3.29 | 0.074 | 8.04 | 0.004 | 8.38 | 0.029 | BO, control in fens |
| *Hyalosphenia minuta* | 0.01 | 0.949 | 26.95 | 0.001 | 9.91 | 0.016 | BO; WLD in BO, CTR in fens |
| *Hyalosphenia papilio* | 9.35 | 0.005 | 6.61 | 0.082 | 26.24 | 0.001 | CTR, more sensitive in fens |
| *Planocarina marginata* | 18.55 | 0.001 | 20.55 | 0.001 | 0.00 | 0.375 | CTR, RF |
| *Nebela collaris* | 0.64 | 0.406 | 30.44 | 0.001 | 0.00 | 0.986 | RF |
| *Nebela tincta* | 2.07 | 0.249 | 57.02 | 0.001 | 14.19 | 0.021 | PF, WLD in fens (not present in bog) |
| *Phryganella arcopodia* | 6.49 | 0.01 | 7.91 | 0.011 | 8.57 | 0.029 | CTR, BO, less sensitive in RF |
| cf. *Phryganella paradoxa* | 1.50 | 0.306 | 27.58 | 0.001 | 20.16 | 0.001 | BO, WLD in BO |
| *Physochila griseola* | 3.91 | 0.064 | 11.03 | 0.005 | 2.69 | 0.177 | RF |
| *Valkanovia elegans* | 6.60 | 0.025 | 16.14 | 0.001 | 4.04 | 0.191 | WLD, BO |
| *Corythion-Trinema* type | 12.58 | 0.001 | 8.92 | 0.008 | 0.21 | 0.881 | WLD, PF |
| Overall functional traits | 22755.36 | 0.001 | 66914.44 | 0.001 | 6727.86 | 0.001 |  |
| Aperture size | 198.30 | 0.001 | 857.03 | 0.001 | 140.50 | 0.013 | Decrease from RF to BO and from CTR to WLD. More sensitive in fens. |
| Biovolume | 22463.46 | 0.001 | 65782.96 | 0.001 | 6486.07 | 0.001 | Decrease from RF to BO and from CTR to WLD. More sensitive in fens.. |
| Mixotrophy | 18.34 | 0.001 | 13.31 | 0.031 | 28.08 | 0.001 | Decrease from CTR to WLD, More sensitive in fens. |
| Aperture position |  |  |  |  |  |  |  |
| Axial | 4.09 | 0.02 | 3.17 | 0.183 | 4.81 | 0.04 | Decrease from CTR to WLD. More sensitive in fens. |
| Acrostomic | 1.72 | 0.215 | 18.22 | 0.005 | 0.04 | 0.973 | RF |
| Plagiostomic | 7.69 | 0.019 | 14.85 | 0.013 | 0.25 | 0.92 | RF |
| Test compression |  |  |  |  |  |  |  |
| Spherical | 3.19 | 0.096 | 2.59 | 0.311 | 5.32 | 0.066 |  |
| Sub spherical | 0.02 | 0.876 | 3.32 | 0.221 | 2.75 | 0.259 |  |
| Compressed | 2.83 | 0.079 | 36.66 | 0.001 | 6.83 | 0.017 | Decrease from CTR to WLD. More sensitive in fens. |
| Strongly compressed | 0.07 | 0.731 | 7.89 | 0.007 | 0.15 | 0.877 | PF |
| Test material |  |  |  |  |  |  |  |
| Protein | 11.20 | 0.003 | 12.66 | 0.016 | 21.20 | 0.002 | Decrease from CTR to WLD.  More sensitive in fens. |
| Silica | 8.76 | 0.016 | 25.86 | 0.006 | 1.51 | 0.61 | Increase from CTR to WLD, prefers fens. |
| Silica + org | 0.01 | 0.84 | 7.94 | 0.004 | 1.40 | 0.156 | PF |
| Calcite | 0.01 | 0.892 | 4.08 | 0.025 | 0.90 | 0.575 |  |
| Recycled idiosomes | 0.08 | 0.755 | 65.32 | 0.001 | 1.97 | 0.316 | Increase along fertility gradient |
| Xenosomes | 6.20 | 0.029 | 6.64 | 0.11 | 1.49 | 0.509 | Increase from WLD to CTR |
| Phylogeny |  |  |  |  |  |  |  |
| Arcellinida | 9.78 | 0.005 | 9.48 | 0.034 | 9.86 | 0.01 | Decrease from CTR to WLD, More sensitive in fens. RF |
| Euglyphida | 6.99 | 0.021 | 23.61 | 0.003 | 1.19 | 0.646 | Increase from CTR to WLD, PF |
| Stramenopiles | 12.62 | 0.001 | 18.85 | 0.001 | 13.56 | 0.001 | Decrease from CTR to WLD.  More sensitive in fens. |

Supplementary Table 6. Abbreviations of taxon names.

| **Abbreviation** | **Taxon** |
| --- | --- |
| ALA MIL | *Alabasta militaris* |
| AMP STE | *Amphitrema stenostoma* |
| AMP WRI | *Amphitrema wrightianum* |
| ARC DIS | *Arcella discoides* |
| ARC FLA | *Archerella flavum* |
| ARC HEM | *Arcella hemisphaerica* |
| ARC VUL | *Arcella vulgaris* |
| ASS MUS | *Assulina muscorum* |
| ASS SEM | *Assulina seminulum* |
| CEN ACU | *Centropyxis acuelata* |
| CEN PLA | *Centropyxis platystoma* |
| COR−TRI | *Corythion-Trinema type* |
| CRY HOR | *Cryptodifflugia horrida* |
| CRY OVI | *Cryptodifflugia oviformis* |
| CRY SAC | *Cryptodifflugia sacculus* |
| CYC EUR | *Cyclopyxis eurystoma* |
| DIF GLO | *Difflugia globulosa* |
| DIF LEI | *Difflugia leidyi* |
| DIF LUC | *Difflugia lucida type* |
| DIF PUL | *Difflugia pulex* |
| EUG CIL | *Euglypha ciliata* |
| EUG COM | *Euglypha compressa* |
| EUG CRI | *Euglypha cristata* |
| EUG PEN | *Euglypha penardi* |
| EUG ROT | *Euglypha rotunda* |
| EUG STR | *Euglypha strigosa* |
| EUG TUB | *Euglypha tuberculata* |
| GAL ART | *Galeripora artocrea* |
| GAL CAT | *Galeripora catinus* |
| HEL PET | *Heleopera petricola* |
| HEL ROS | *Heleopera rosea* |
| HEL SPH | *Heleopera sphagni* |
| HEL SYL | *Heleopera sylvatica* |
| HYA ELE | *Hyalosphenia elegans* |
| HYA MIN | *Hyalosphenia minuta* |
| HYA PAP | *Hyalosphenia papilio* |
| HYA SUB | *Hyalosphenia subflava* |
| LES SPI | *Lesquereusia spiralis* |
| NEB COL | *Nebela collaris* |
| NEB TIN | *Nebela tincta* |
| PHR ACR | *Phryganella arcopodia* |
| PHR PAR | *cf. Phryganella paradoxa* |
| PHY GRI | *Physochila griseola* |
| PLA MAR | *Planocarina marginata* |
| PLA SPI | *Placocista spinosa* |
| PSE FAS | *Pseudodifflugia fascicularis* |
| PSE FUL | *Pseudodifflugia fulva* |
| SPH LEN | *Sphenoderia lenta* |
| TRA DEN | *Tracheleuglypha dentata* |
| TRI ARC | *Trigonopyxis arcula* |
| TRI ENC | *Trinema enchelys* |
| VAL ELE | *Valkanovia elegans* |

Supplementary table 7. The explaining power of different functional traits in the NMDS analysis (Figure 2).

| **Functional trait** | | **r^2^** | **p-value** |
| --- | --- | --- | --- |
| Aperture size | | 0.54 | 0.001 |
| Biovolume | | 0.75 | 0.001 |
| Mixotrophy | | 0.58 | 0.001 |
| Aperture position |  |  |  |
|  | Axial | 0.09 | 0.110 |
|  | Acrostomic | 0.42 | 0.001 |
|  | Plagiostomic | 0.45 | 0.001 |
| Test compression |  |  |  |
|  | Spherical | 0.01 | 0.808 |
|  | Sub spherical | 0.08 | 0.112 |
|  | Compressed | 0.50 | 0.001 |
|  | Strongly compressed | 0.14 | 0.020 |
| Test material |  |  |  |
|  | Protein | 0.46 | 0.001 |
|  | Silica | 0.53 | 0.001 |
|  | Silica + org | 0.06 | 0.234 |
|  | Calcite | 0.08 | 0.112 |
|  | Recycled idiosomes | 0.65 | 0.001 |
|  | Xenosomes | 0.31 | 0.001 |
| Phylogeny |  |  |  |
|  | Arcellinida | 0.29 | 0.001 |
|  | Euglyphida | 0.5 | 0.001 |
|  | Stramenopiles | 0.72 | 0.001 |

Supplementary Table 8. The explaining power of different environmental variables and vegetation types in the NMDS analysis (Figure 3c).

|  | **r^2^** | **p-value** |
| --- | --- | --- |
| pH | 0.77 | 0.001 |
| N | 0.42 | 0.001 |
| Ca | 0.77 | 0.001 |
| Fe | 0.35 | 0.001 |
| Mg | 0.63 | 0.001 |
| P | 0.54 | 0.001 |
| S | 0.37 | 0.001 |
| SphagnumWater | 0.32 | 0.001 |
| WT | 0.51 | 0.001 |
| Shade | 0.72 | 0.001 |
| T5 | 0.20 | 0.006 |
| T15 | 0.46 | 0.001 |
| Other mosses | 0.23 | 0.004 |
| *Sphagnum* | 0.10 | 0.084 |
| Shrubs and trees | 0.17 | 0.006 |
| Cyperaceae | 0.16 | 0.014 |
| Herbs | 0.24 | 0.002 |
| Grasses | 0.04 | 0.259 |

Supplementary Table 9: Tukey multiple comparisons of means of functional traits with 95% family-wise confidence level. RF = rich fen, PF = poor fen, BO = bog, WLD = water level drawdown, CTR = control

| **Functional trait** | | **Site*Treatment** | **diff** | **lwr** | **upr** | **p-value** |
| --- | --- | --- | --- | --- | --- | --- |
| Aperture size | | RF WLD-RF CTR | -162.53 | -306.18 | -18.88 | 0.018 |
|  |  | PF WLD-PF CTR | -124.33 | -259.77 | 11.11 | 0.089 |
|  |  | BO WLD-BO CTR | 22.97 | -109.04 | 154.98 | 0.995 |
| Biovolume | | RF WLD-RF CTR | -8146.92 | -1836.27 | -457.58 | 0.000 |
|  |  | PF WLD-PF CTR | -2438.73 | -5917.09 | 1039.61 | 0.314 |
|  |  | BO WLD-BO CTR | -315.13 | -3705.41 | 3075.14 | 1.000 |
| Mixotrophy | | RF WLD-RF CTR | -2.51 | -4.89 | -0.12 | 0.034 |
|  |  | PF WLD-PF CTR | -3.76 | -6.01 | -1.52 | 0.000 |
|  |  | BO WLD-BO CTR | -0.02 | -2.21 | 2.17 | 1.000 |
| Aperture position | Axial | RF WLD-RF CTR | -3.99 | -9.59 | 1.60 | 0.294 |
|  |  | PF WLD-PF CTR | -5.18 | -10.45 | 0.100 | 0.057 |
|  |  | BO WLD-BO CTR | 1.22 | -3.92 | 6.35 | 0.981 |
|  | Acrostomic | RF WLD-RF CTR | -0.77 | -3.12 | 1.56 | 0.921 |
|  |  | PF WLD-PF CTR | -0.52 | -2.72 | 1.68 | 0.981 |
|  |  | BO WLD-BO CTR | -0.41 | -2.55 | 1.74 | 0.993 |
|  | Plagiostomic | RF WLD-RF CTR | 1.77 | -1.71 | 5.25 | 0.657 |
|  |  | PF WLD-PF CTR | 2.04 | -1.24 | 5.32 | 0.446 |
|  |  | BO WLD-BO CTR | 0.8 | -2.4 | 3.99 | 0.976 |
| Test material | Protein | RF WLD-RF CTR | -2.18 | -4.84 | 0.48 | 0.166 |
|  |  | PF WLD-PF CTR | -4.65 | -7.16 | -2.14 | 0.000 |
|  |  | BO WLD-BO CTR | 0.75 | -1.69 | 3.20 | 0.941 |
|  | Silica | RF WLD-RF CTR | 4.3 | -1.34 | 9.95 | 0.228 |
|  |  | PF WLD-PF CTR | 3.00 | -2.32 | 8.32 | 0.554 |
|  |  | BO WLD-BO CTR | 0.68 | -4.51 | 5.86 | 0.999 |
|  | Silica + org | RF WLD-RF CTR | -0.5 | -1.73 | 0.74 | 0.836 |
|  |  | PF WLD-PF CTR | -0.31 | -1.47 | 0.85 | 0.967 |
|  |  | BO WLD-BO CTR | 0.59 | -0.54 | 1.72 | 0.633 |
|  | Calcite | RF WLD-RF CTR | 0.14 | -1.14 | 1.41 | 1.000 |
|  |  | PF WLD-PF CTR | -0.37 | -1.57 | 0.84 | 0.944 |
|  |  | BO WLD-BO CTR | 0.13 | -1.04 | 1.30 | 0.999 |
|  | Recycled idiosomes | RF WLD-RF CTR | -1.24 | -2.95 | 0.46 | 0.274 |
|  |  | PF WLD-PF CTR | 0.67 | -0.93 | 2.28 | 0.812 |
|  |  | BO WLD-BO CTR | 0.05 | -1.51 | 1.62 | 1.000 |
|  | Xenosomes | RF WLD-RF CTR | -2.63 | -6.57 | 1.32 | 0.371 |
|  |  | PF WLD-PF CTR | -2.00 | -5.73 | 1.72 | 0.603 |
|  |  | BO WLD-BO CTR | -0.6 | -4.23 | 3.03 | 0.996 |
| Test compression | Spherical | RF WLD-RF CTR | 0.44 | -0.83 | 1.71 | 0.905 |
|  |  | PF WLD-PF CTR | 0.59 | -0.61 | 1.78 | 0.696 |
|  |  | BO WLD-BO CTR | 0.03 | -1.14 | 1.19 | 1.000 |
|  | Sub-spherical | RF WLD-RF CTR | 1.66 | -2.10 | 5.42 | 0.777 |
|  |  | PF WLD-PF CTR | -0.98 | -4.53 | 2.57 | 0.962 |
|  |  | BO WLD-BO CTR | -0.77 | -4.22 | 2.69 | 0.986 |
|  | Compressed | RF WLD-RF CTR | -3.99 | -9.08 | 1.10 | 0.203 |
|  |  | PF WLD-PF CTR | -3.64 | -8.44 | 1.16 | 0.234 |
|  |  | BO WLD-BO CTR | 2.02 | -2.66 | 6.70 | 0.793 |
|  | Strongly compressed | RF WLD-RF CTR | -0.22 | -2.92 | 2.48 | 1.000 |
|  |  | PF WLD-PF CTR | 0.38 | -2.17 | 2.92 | 0.998 |
|  |  | BO WLD-BO CTR | 0.32 | -2.16 | 2.80 | 0.999 |
| Phylogeny | Arcellinida | RF WLD-RF CTR | -5.60 | -10.54 | -0.66 | 0.018 |
|  |  | PF WLD-PF CTR | -5.43 | -10.07 | -0.77 | 0.014 |
|  |  | BO WLD-BO CTR | 1.24 | -3.30 | 5.77 | 0.965 |
|  | Euglyphida | RF WLD-RF CTR | 4.21 | -1.71 | 10.13 | 0.299 |
|  |  | PF WLD-PF CTR | 2.89 | -2.69 | 8.47 | 0.641 |
|  |  | BO WLD-BO CTR | 0.91 | -4.53 | 6.35 | 0.996 |
|  | Stramenopiles | RF WLD-RF CTR | -1.81 | -3.18 | -0.44 | 0.004 |
|  |  | PF WLD-PF CTR | -1.31 | -2.61 | -0.02 | 0.045 |
|  |  | BO WLD-BO CTR | -0.54 | -1.80 | 0.72 | 0.798 |


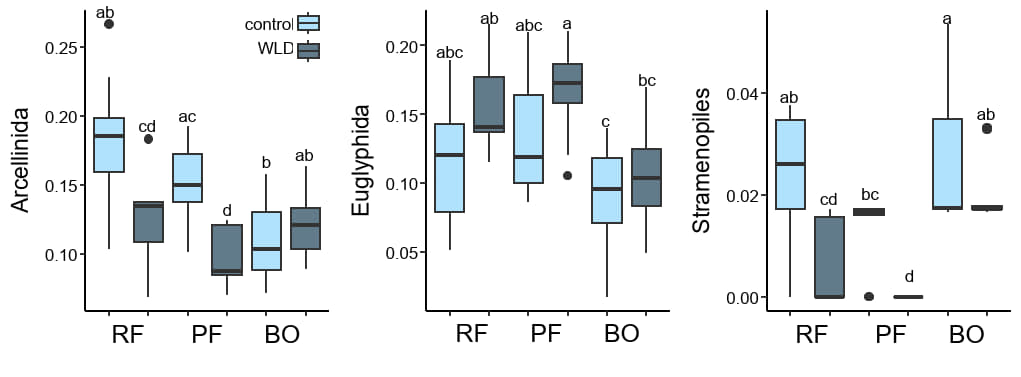


Supplementary Figure 3: Phylogeny of testate amoebae expressed as community weighted mean in the six study areas. The letters indicate significant differences in the pairwise comparison. RF = Rich fen, PF = Poor fen, BO = Bog.


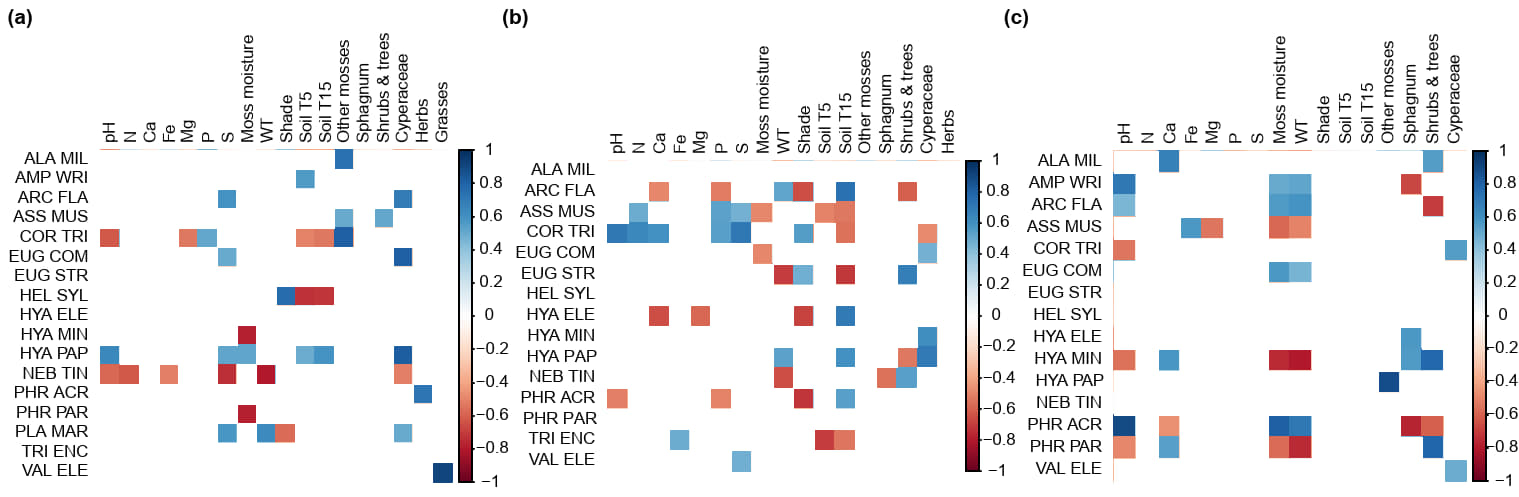


Supplementary Figure 4. Fourth corner analysis illustrating correlations between environmental variables and selected taxa in **(a)** rich fen, **(b)** poor fen, and **(c)** bog. Red indicates negative correlation and blue positive correlation. Only statistically significant (p-value < 0.05) correlations are shown.


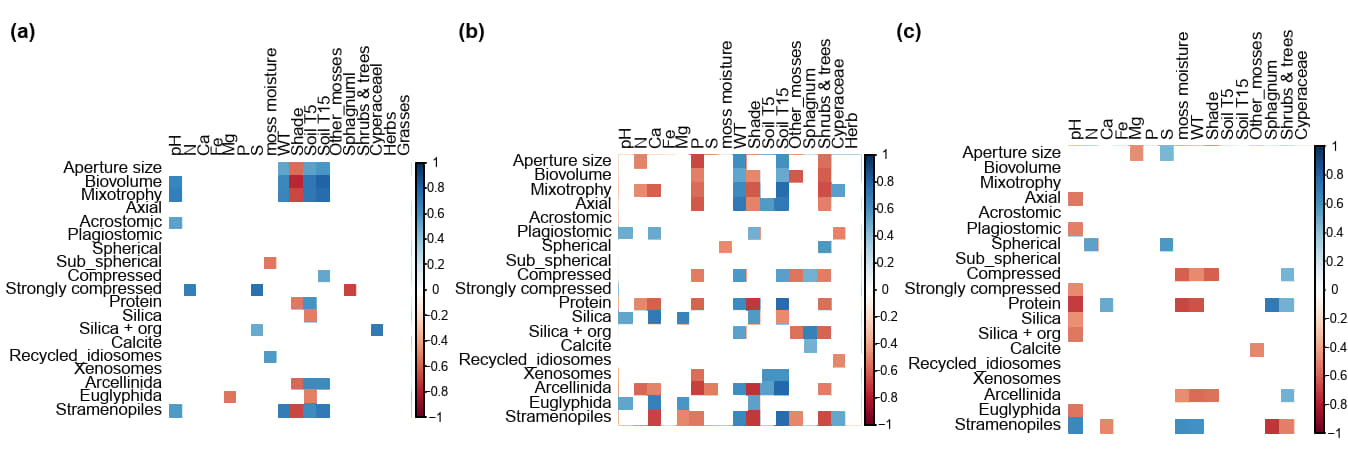


Supplementary Figure 5. Fourth corner analysis illustrating correlations between environmental variables and selected functional traits in **(a)** rich fen, **(b)** poor fen, and **(c)** bog. Red indicates negative correlation and blue positive correlation. Only statistically significant (p-value < 0.05) correlations are shown.

References

1. Köster, E., Chapman, J. P. B., Barel, J. M., Korrensalo, A., Laine, A. M., Vasander, H. T., & Tuittila, E. (2023). Water level drawdown makes boreal peatland vegetation more responsive to weather conditions. *Global Change Biology*, **29**(19), 5691–5705. https://doi.org/10.1111/gcb.16907
2. Kuuri-Riutta, O., Le Geay, M., Jassey, V.E.J., Barel, J.M., Laine, A.M., Ylänne, H. and Tuittila, E.-S. (2025). Microbial and bryospheric photosynthesis of boreal peatlands have peatland-type-specific responses to long-term drying. *New Phytologist*.  <https://doi.org/10.1111/nph.70519>
3. Kokkonen, N. A. K., Laine, A. M., Laine, J., Vasander, H., Kurki, K., Gong, J., Tuittila, E.–S., & Collins, B. (2019). Responses of peatland vegetation to 15-year water level drawdown as mediated by fertility level. *Journal of vegetation science,* 30(6), 1206–1216. <https://doi.org/10.1111/jvs.12794>
